# Methylome and transcriptome analyses of soybean response to bean pyralid larvae

**DOI:** 10.1186/s12864-021-08140-w

**Published:** 2021-11-18

**Authors:** Wei-Ying Zeng, Yu-Rong Tan, Sheng-Feng Long, Zu-Dong Sun, Zhen-Guang Lai, Shou-Zhen Yang, Huai-Zhu Chen, Xia-Yan Qing

**Affiliations:** Guangxi Academy of Agricultural Sciences, Nanning, 530007 Guangxi China

**Keywords:** Soybean, Bean pyralid, DNA methylation, Differentially methylated genes, Gene expression

## Abstract

**Background:**

Bean pyralid is one of the major leaf-feeding insects that affect soybean crops. DNA methylation can control the networks of gene expressions, and it plays an important role in responses to biotic stress. However, at present the genome-wide DNA methylation profile of the soybean resistance to bean pyralid has not been reported so far.

**Results:**

Using whole-genome bisulfite sequencing (WGBS) and RNA-sequencing (RNA-seq), we analyzed the highly resistant material (Gantai-2-2, HRK) and highly susceptible material (Wan82–178, HSK), under bean pyralid larvae feeding 0 h and 48 h, to clarify the molecular mechanism of the soybean resistance and explore its insect-resistant genes. We identified 2194, 6872, 39,704 and 40,018 differentially methylated regions (DMRs), as well as 497, 1594, 9596 and 9554 differentially methylated genes (DMGs) in the HRK0/HRK48, HSK0/HSK48, HSK0/HRK0 and HSK48/HRK48 comparisons, respectively. Through the analysis of global methylation and transcription, 265 differentially expressed genes (DEGs) were negatively correlated with DMGs, there were 34, 49, 141 and 116 negatively correlated genes in the HRK0/HRK48, HSK0/HSK48, HSK0/HRK0 and HSK48/HRK48, respectively. The MapMan cluster analysis showed that 114 negatively correlated genes were clustered in 24 pathways, such as protein biosynthesis and modification; primary metabolism; secondary metabolism; cell cycle, cell structure and component; RNA biosynthesis and processing, and so on. Moreover, CRK40; CRK62; STK; MAPK9; L-type lectin-domain containing receptor kinase VIII.2; CesA; CSI1; fimbrin-1; KIN-14B; KIN-14 N; KIN-4A; cytochrome P450 81E8; BEE1; ERF; bHLH25; bHLH79; GATA26, were likely regulatory genes involved in the soybean responses to bean pyralid larvae. Finally, 5 DMRs were further validated that the genome-wide DNA data were reliable through PS-PCR and 5 DEGs were confirmed the relationship between DNA methylation and gene expression by qRT-PCR. The results showed an excellent agreement with deep sequencing.

**Conclusions:**

Genome-wide DNA methylation profile of soybean response to bean pyralid was obtained for the first time. Several specific DMGs which participated in protein kinase, cell and organelle, flavonoid biosynthesis and transcription factor were further identified to be likely associated with soybean response to bean pyralid. Our data will provide better understanding of DNA methylation alteration and their potential role in soybean insect resistance.

**Supplementary Information:**

The online version contains supplementary material available at 10.1186/s12864-021-08140-w.

## Background

Bean pyralid (*Lamprosema indicata* (Fabricius)) is an important leaf-feeding insect, is widely distributed throughout the world and is found in China, Korea, Japan, India, the Americas and Africa [1]. During the years when serious damage has occurred, it can produce 4 to 5 generations a year, with overlapping of generations in the soybean producing areas. Bean pyralid larva spins or wraps leaves to form wrapped leaves and hide in them. After feeding on leaves, only veins and petioles remain, which leads to the difficulty in the normal photosynthesis of soybean and affects plant growth, in turn leading to serious yield losses [2, 3]. In view of the serious damages caused by bean pyralid, the highly resistant and highly susceptible soybeans were identified [3]. The resistance of soybean to bean pyralid is inherited by two pairs of major genes plus polygenes, and the resistance loci are mainly located in linkage groups A2, C2, D1a, D1b, H, K and O [4–6]. The contents of soluble sugar, JA, CAT and PPO are related to the induction of bean pyralid larvae. Meanwhile, the contents of SOD, ET and ABA are related to the pest induction and genotypes [7]. Trypsin inhibitor A-like; chalcone isomerase 4-like; lipoxygenase-9; alpha-dioxygenase 1-like; lectin precursor; peroxidase 12-like; stress-induced protein SAM22; and so on, may be the potential target proteins (genes) for soybean to resist bean pyralid larvae [8–10]. In addition, such miRNAs as gma-miR156q; gma-miR166u; gma-miR166b; gma-miR319d; gma-miR394a-3p; and gma-miR396e, may also participate in the regulation of soybean resistance to bean pyralid larvae [11]. However, very little is known regarding the mechanism of epigenetic regulation related to soybean resistance to bean pyralid.

DNA methylation can turn off the activities of some genes, while demethylation can induce gene reactivity and expression. In addition, it can control the networks of gene expressions, thereby playing an important role in plant growth, development, and responses to biotic stress, and is an important means of regulating genome function [12]. Plants are often attacked by pathogens and pests during their growth and development processes. Such attacks can induce the plants to produce physiological or even gene level variations and changes in gene expressions in order to avoid or endure adversity. However, the main research involves the epigenetic effects of biotic stress on plants undergoing disease stress, such as xanthomonas oryzae pv.oryzae [13, 14]; tobacco mosaic virus [15, 16]; soybean cyst nematode [17] and arabidopsis cyst nematode [18]. There have been few studies conducted regarding epigenetic inheritance caused by insect. Therefore, DNA methylation can be used as an entry point to explore soybean resistance to bean pyralid.

In this study, we performed methylome and transcriptome analyses to different insect resistant material in soybean. We used the leaves of Gantai-2-2 (highly resistant material) and Wan82–178 (highly susceptible material) [3] before and after exposure to bean pyralid larvae as the experimental materials. This is the first time to deepen the understanding of the regulatory relationship between DNA methylation and gene expression in soybean undergoing insect stress, to explore the role of related genes in soybean resistance to bean pyralid larvae, and how gene expression is regulated in the whole genome of the soybean resistance to bean pyralid larvae, so as to lay a foundation for further research regarding the molecular mechanism of soybean response to insect stress at the epigenetic level.

## Results

### Bisulfite sequencing of genomes of different soybean cultivars

To study the characteristics and patterns of DNA methylation in the leaves of different insect resistant materials, we used the Illumian HiSeq4000 platform to construct DNA libraries of the highly resistant material (Gantai-2-2, HRK) and highly susceptible material (Wan82–178, HSK), respectively. The leaves were subjected to bean pyralid larvae feeding for 0 h and 48 h. The results showed that each sample produced 40 G filtered clean bases on average, the Q20 were 98.00, 97.99, 98.14 and 98.30%, respectively (Table 1). Meanwhile, more than 99.50% cytosines were converted, which indicated that the adopted high-throughput sequencing technology had a high recognition rate (Table 1). There were 89.57, 89.92, 92.09 and 90.50% clean reads were mapped to the reference soybean genome of Glycine_max_v2.0 (https://www.ncbi.nlm.nih.gov/assembly/GCF_000004515.4) (Table 1). The comparison results indicated that DNA methylation sequencing conversion rate was high and sequencing quality was qualified.

**Table 1:** Summary of WGBS data

Sample ID	Q20 Rate (%)	Clean Reads Number	Clean Rate (%)	Mapping Rate (%)	Uniquely Mapping Rate (%)	Bisulfite Conversion Rate (%)	Duplication Rate (%)	Average Depth (×)	1 × Reads Coverage (%)
HRK0	98.00	266,666,670	93.61	89.57	75.23	99.60	16.21	25.45	93.61
HRK48	97.99	266,666,668	93.21	89.92	74.59	99.58	16.16	25.22	93.43
HSK0	98.14	266,666,670	87.64	92.09	76.98	99.61	15.42	26.28	93.54
HSK48	98.30	266,666,668	88.29	90.50	75.76	99.58	17.14	25.30	93.61

Note: HRK represented the highly resistant marterial Gantai-2-2; HSK represented the highly susceptible marterial Wan82–178; and the numbers 0 and 48 represented the processing times

In addition, to estimate whether or not the sequencing depth could satisfy the coverage of the sequencing data, the sequencing coverages of four DNA libraries were counted. Then, when the sequencing depth was 25×, it was considered that more than 93.40% reading segments had been successfully covered (Table 1). Therefore, it was inferred that the overall sequencing quality of the four DNA libraries was relatively good and the vast majority of base sites had been covered.

To enhance the current understanding of the epigenetic regulation and DNA methylation levels of soybean leaves with different resistance to bean pyralid larvae, we further compared the genome-wide methylation levels of the four samples. It was determined that methylated cytosine ranged from 18.37 to 21.30%. Meanwhile, the average level of methylated cytosine in each context was also calculated, in the CG context ranged from 68.27 to 74.71%; in the CHG context ranged from 42.15 to 47.64%; and in the CHH context ranged from 4.90 to 5.81% (Table 2). It was observed that DNA methylation level was highest in the CG context, and lower in the CHG and CHH contexts. These findings indicated that the CG context was the most important methylation context for soybean. There were significant differences in DNA methylation levels among the different resistance materials. For example, the DNA methylation levels of the resistant material were lower than those of the susceptible material. Also, the DNA methylation levels of the resistant material increased while those of the susceptible material decreased after insect feeding stress. The increasing and decreasing results obtained in our study were similar to the inconsistent variations of methylation observed in different types of rice undergoing saline-alkali stress [19].

**Table 2:** The average methylation level of methylation and some elements in different contexts

Context	Sample	Average Level of Methylation (%)	CDS (%)	Down2k (%)	Up2k (%)	Exon (%)	mRNA (%)	Repeat (%)	CpG-island (%)
C	HRK0	19.84	5.207	10.132	11.062	5.299	7.303	25.815	28.597
	HRK48	21.30	5.441	11.182	12.218	5.607	7.965	27.090	28.950
	HSK0	20.96	5.563	11.249	12.378	5.677	8.019	26.182	28.698
	HSK48	18.37	5.156	8.924	9.688	5.100	7.008	25.396	26.625
CG	HRK0	72.36	18.791	36.162	36.711	17.625	33.092	94.113	62.019
	HRK48	73.33	18.121	37.534	38.155	17.301	33.034	94.326	61.385
	HSK0	74.71	19.587	39.442	40.439	18.570	34.679	94.593	62.283
	HSK48	68.27	19.732	31.538	31.641	17.581	32.772	93.837	57.562
CHG	HRK0	46.14	7.715	25.111	28.541	9.231	12.804	70.380	48.399
	HRK48	47.64	8.012	26.576	30.084	9.579	13.621	70.935	48.132
	HSK0	47.40	8.127	27.020	31.062	9.665	13.959	69.872	47.921
	HSK48	42.15	7.190	21.644	24.666	8.485	11.970	68.763	43.650
CHH	HRK0	4.90	1.318	3.008	3.525	1.460	1.942	6.923	6.062
	HRK48	5.81	1.524	3.587	4.201	1.696	2.353	8.063	6.968
	HSK0	5.08	1.364	3.154	3.686	1.511	2.064	6.939	6.056
	HSK48	5.29	1.427	2.961	3.406	1.567	2.097	7.897	6.582

**Fig. 1:**
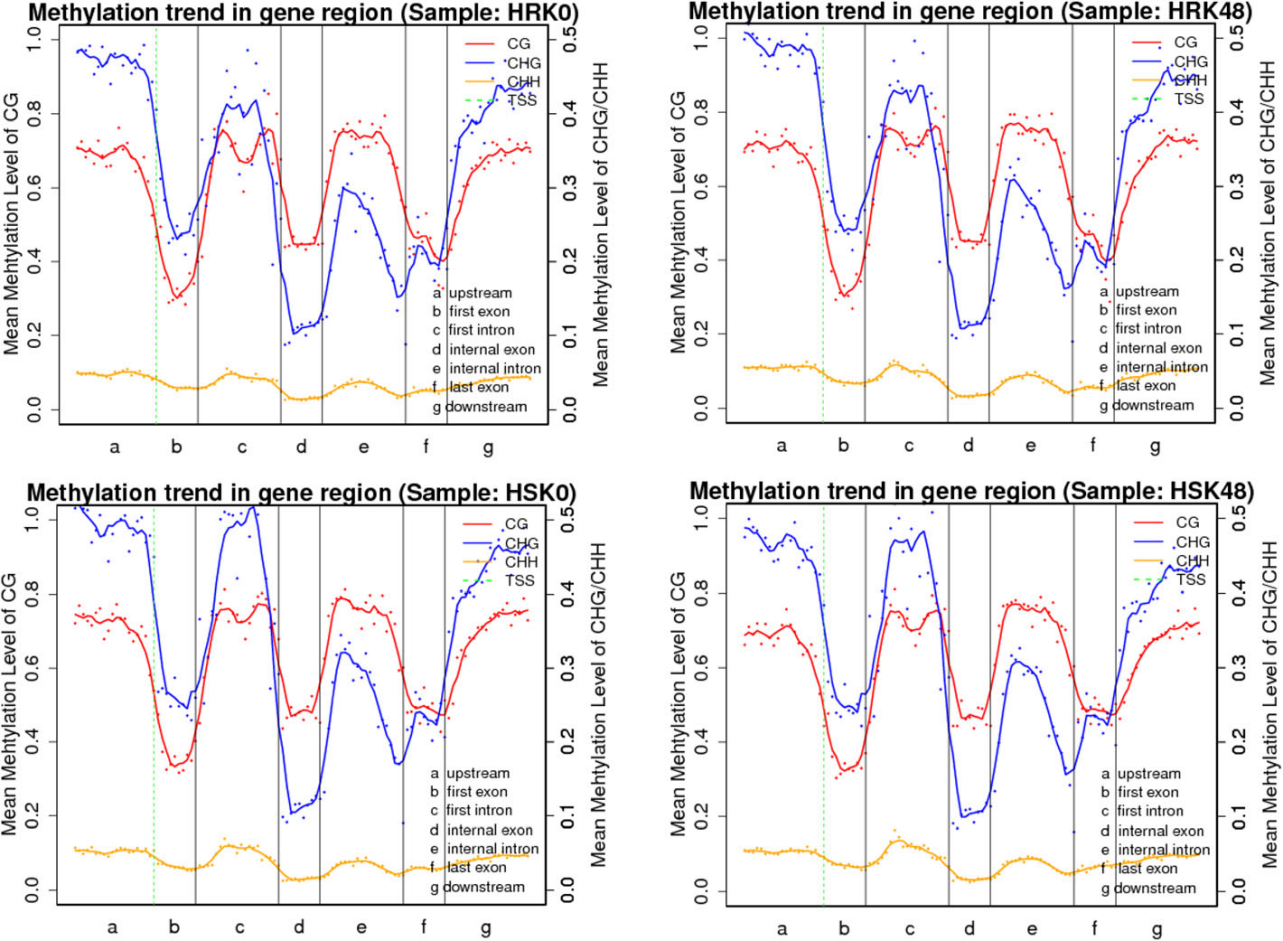
Canonical DNA methylation profiles of the entire transcriptional units

To investigate the DNA methylation patterns in different soybean genomic regions, we analyzed the methylation profiles in gene regions (Table 2). It was observed that DNA methylation level was highest in the CG context followed by CHG and CHH contexts in each gene region of the four samples. Repeat and CpG-island regions had the highest methylation levels of the different gene regions, which indicated that these two regions may be epigenetic regulatory regions which alter gene expressions. Meanwhile, the methylation levels were much higher in mRNA than in exon. After bean pyraild larvae feeding, the average methylation levels of the CG, CHG and CHH contexts in the resistant material were increased among the different transcription regions. However, the opposite effects were observed in the susceptible material. In the CG, CHG and CHH contexts, the methylation levels were more abundant in upstream and downstream regions than in exon regions (Fig.1). In addition, methylation was more frequent in the CHG context than in the CG and CHH contexts in upstream, first intron, and downstream regions, which indicated that there were some tendencies and differences among the different transcription regions.

### Distribution ratios of the mCG, mCHG, and mCHH in the methylated C-base

To further analyze the distributions of the different methylated sites, all of the methylated C sites were selected and recombined (Table 3). After bean pyralid larvae feeding for 48 h, in the resistant material, mCG decreased from 36.16 to 34.47%, mCHG decreased from 34.42 to 32.80% and mCHH increased from 29.42 to 32.73%. In the susceptible material, mCG decreased from 36.20 to 34.78%, mCHG decreased from 34.22 to 32.86% and mCHH increased from 29.58 to 32.39%. The methylation levels of mCG, mCHG and mCHH in the resistant and susceptible materials were similar before and after bean pyralid larvae feeding. Therefore, the results indicated that the genotype had little effect on the methylation levels of the mCG, mCHG and mCHH, and the methylation levels were mainly affected by insect stress.

**Table 3:** Proportion of CG, CHG and CHH in all methyl-cytosine

Sample		mCG	mCHG	mCHH
HRK0	mC number	17,487,188	16,644,623	14,228,044
	Proportion (%)	36.16	34.42	29.42
HRK48	mC number	17,244,372	16,403,662	16,368,103
	Proportion (%)	34.47	32.80	32.73
HSK0	mC number	17,509,047	16,554,658	14,308,471
	Proportion (%)	36.20	34.22	29.58
HSK48	mC number	17,606,421	16,649,935	16,413,930
	Proportion (%)	34.78	32.86	32.39

**Fig. 2:**
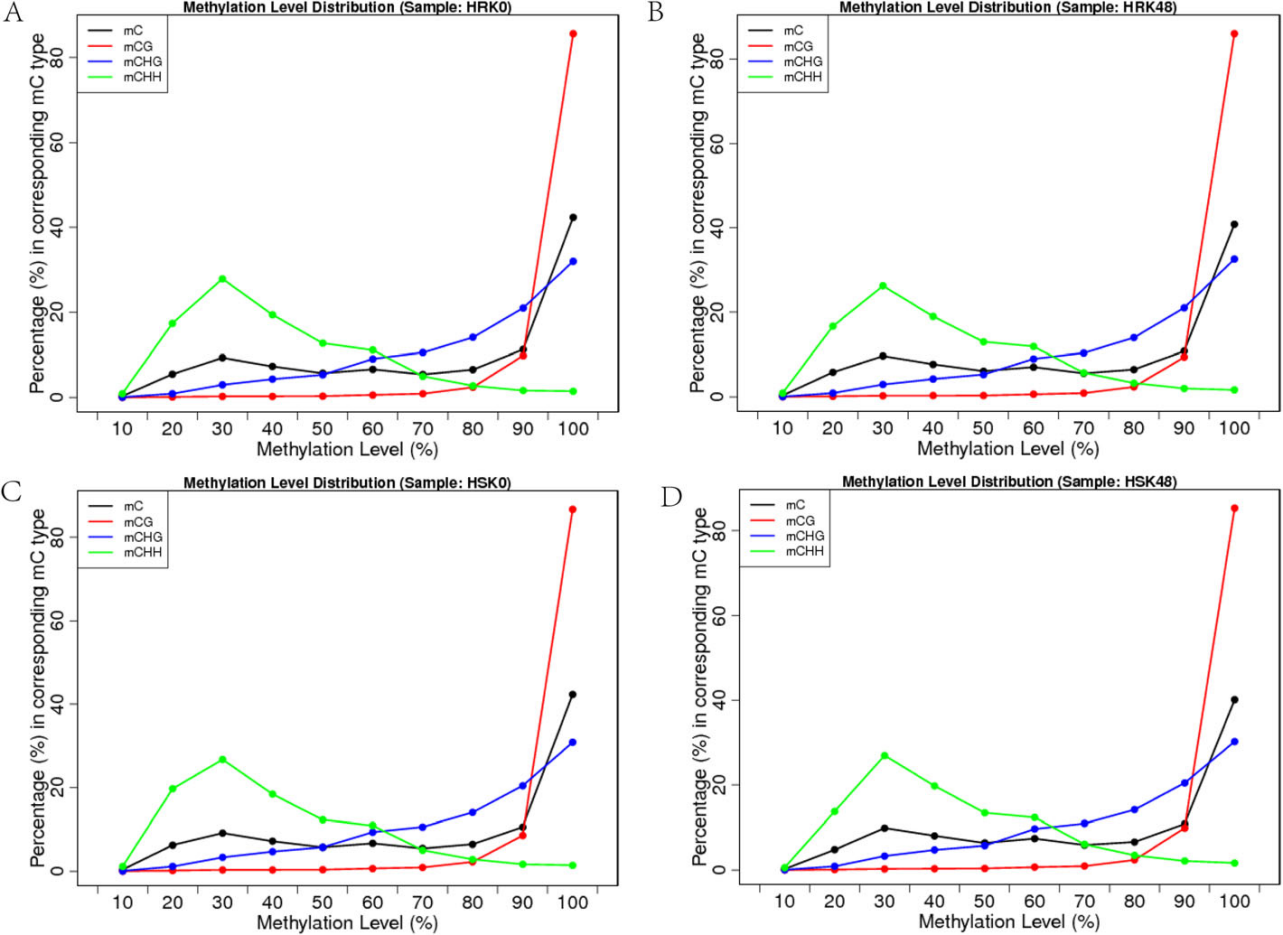
Distribution of methylation level of mC in each sequence context

The methylation levels of each type of methylated C were statistically analyzed (Fig.2). The analysis results revealed that when the methylation levels ranged from 0 to 60%, the distribution proportions of the methylated C followed the order of mCHH > mCHG > mCG. However, when the methylation levels were between 60 and 80%, the distribution proportions followed the order of mCHG > mCHH > mCG. Furthermore, when the methylation levels were higher than 80%, the mCG site was significantly increased.

### Identification of the differentially methylated genes

To study the differential methylation among different resistant soybean varieties, we successfully identified the differentially methylated regions (DMRs). The number of DMRs identified in HRK0/HRK48 in the CG, CHG and CHH contexts were 664, 1550 and 0, respectively; in HSK0/HSK48 were 2200, 4670 and 2, respectively; in HSK0/HRK0 were 19,200, 20,272 and 31, respectively; and in HSK48/HRK48 were 19,178, 20,807 and 33, respectively. Also, DMRs detected at the CG and CHG contexts were significantly higher than that at the CHH context.

We divided the DMRs into the following two groups: DMR-associated genes (DMGs) and DMR-associated promoters (DMPs), which DMRs overlapped with the genes and promoters [20]. In HRK0/HRK48, 497 no-repeated DMGs (207 hyper-DMGs and 290 hypo-DMGs) and 223 no-repeated DMPs (99 hyper-DMPs and 124 hypo-DMPs) were identified, of which 48 DMGs appeared in both promoter regions and gene bodies, simultaneously. In HSK0/HSK48, 1594 no-repeated DMGs (687 hyper-DMGs, 882 hypo-DMGs and 25 shared-DMGs) and 612 no-repeated DMPs (235 hyper-DMPs and 377 hypo-DMPs) were identified, of which 186 DMGs appeared in both promoter regions and gene bodies, simultaneously. In HSK0/HRK0, 9596 no-repeated DMGs (2717 hyper-DMGs, 6577 hypo-DMGs and 302 shared-DMGs) and 3173 no-repeated DMPs (1357 hyper-DMPs, 1786 hypo-DMPs and 30 shared-DMPs) were identified, of which 1479 DMGs appeared in both promoter regions and gene bodies, simultaneously. In HSK48/HRK48, 9554 no-repeated DMGs (2944 hyper-DMGs, 6302 hypo-DMGs and 308 shared-DMGs) and 3217 no-repeated DMPs (1542 hyper-DMPs, 1636 hypo-DMPs and 39 shared-DMPs) were identified, of which 1379 DMGs appeared in both promoter regions and gene bodies, simultaneously. In HRK0/HRK48, HSK0/HSK48, HSK0/HRK0 and HSK48/HRK48, there were more hypo-DMGs and hypo-DMPs than hyper-DMGs and hyper-DMPs. Therefore, it was confirmed that the decreases of genome-wide DNA methylation levels may be one of the causes of the responses of plants to insect stress.

### Gene ontology (GO) annotation and Kyoto encyclopedia of genes and genomes (KEGG) pathway enrichment analyses of DMGs

To better understand the functions of DMGs, GO annotation analysis was conducted. DMGs in the four comparisons were involved in the biological process, cellular component and molecular function. During the biological process, DMGs were mainly enriched in cellular process, metabolic process, biological regulation, regulation of biological process and response to stimulus. In the cellular component, DMGs were mainly concentrated in the cell, cell part, membrane, membrane part and organelle. In the molecular function, DMGs were mainly involved in binding and catalytic activity. It was speculated that the methylation patterns of the different contexts among the four comparisons were consistent in subcellular localization, molecular function, and biological process.

To further identify the metabolic pathways and functions of DMGs, the obtained DMGs were compared in the KEGG database (Table 4). In HRK0/HRK48, 151 DMGs at the CG context had participated in 71 pathways, with one pathway being significantly enriched; 259 DMGs at the CHG context had participated in 80 pathways, with one pathway being significantly enriched. In HSK0/HSK48, 671 DMGs at the CG context had participated in 108 pathways, with 8 pathways being significantly enriched; 667 DMGs at the CHG context had participated in 103 pathways, with 3 pathways being significantly enriched. In HSK0/HRK0, 6923 DMGs at the CG context had participated in 132 pathways, with 5 pathways being significantly enriched; 1907 DMGs at the CHG context had participated in 134 pathways, with 11 pathways being significantly enriched. In HSK48/HRK48, 6851 DMGs at the CG context had participated in 134 pathways, with 11 pathways being significantly enriched; 1943 DMGs at the CHG context had participated in 125 pathways, with 2 pathways being significantly enriched. Therefore, it was speculated that the methylation of the different contexts may have had a tendency to participate in the regulation of the biological functions. These pathways provided a useful reference for studying the biological processes and functions of the genes.

**Table 4:** Pathway analysis of DMGs

Sample	Site	Pathway	DMGs with Pathway Annotation	*p*-value	Pathway ID
HRK0/HRK48	CG	Fructose and mannose metabolism	3 (1.99%)	0.04756201	ko00051
	CHG	Terpenoid backbone biosynthesis	6 (2.32%)	0.003661496	ko00900
HSK0/HSK48	CG	Basal transcription factors	9 (1.34%)	0.001368397	ko03022
		Fatty acid metabolism	9 (1.34%)	0.0047815	ko01212
		Fatty acid biosynthesis	6 (0.89%)	0.01282847	ko00061
		Propanoate metabolism	6 (0.89%)	0.01282847	ko00061
		Taurine and hypotaurine metabolism	3 (0.45%)	0.0241165	ko00430
		Thiamine metabolism	4 (0.60%)	0.02645737	ko00730
		Vitamin B6 metabolism	5 (0.75%)	0.04115753	ko00750
		Lysine degradation	8 (1.19%)	0.04184757	ko00310
	CHG	Thiamine metabolism	5 (0.75%)	0.005305703	ko00730
		Non-homologous end-joining	2 (0.30%)	0.01649522	ko03450
		mRNA surveillance pathway	23 (3.45%)	0.03234759	ko03015
HSK0/HRK0	CG	Proteasome	62 (0.90%)	3.446782e-07	ko03050
		Porphyrin and chlorophyll metabolism	39 (0.56%)	0.0003851172	ko00860
		Arginine biosynthesis	30 (0.43%)	0.0005153445	ko00220
		Other types of O-glycan biosynthesis	28 (0.40%)	0.0005550764	ko00514
		Inositol phosphate metabolism	67 (0.97%)	0.001972833	ko00562
		Butanoate metabolism	24 (0.35%)	0.005140221	ko03040
		Spliceosome	181 (2.61%)	0.005140221	ko03040
		Purine metabolism	76 (1.10%)	0.006294239	ko00230
		Non-homologous end-joining	6 (0.09%)	0.009701395	ko03450
		Phosphatidylinositol signaling system	62 (0.90%)	0.01495655	ko04070
		2-Oxocarboxylic acid metabolism	50 (0.72%)	0.01933169	ko01210
		Fructose and mannose metabolism	49 (0.71%)	0.02097422	ko00051
		Fatty acid metabolism	43 (0.62%)	0.03164016	ko01212
		Propanoate metabolism	24 (0.35%)	0.03299536	ko00640
		Glycosphingolipid biosynthesis-lacto and neolacto series	3 (0.04%)	0.03962215	ko00601
		Lysine biosynthesis	14 (0.20%)	0.04167333	ko00300
	CHG	Arginine biosynthesis	10 (0.52%)	0.01543515	ko00220
		Histidine metabolism	7 (0.37%)	0.02595471	ko00340
		Benzoxazinoid biosynthesis	4 (0.21%)	0.02953415	ko00402
		Oxidative phosphorylation	25 (1.31%)	0.04560989	ko00190
		Protein export	11 (0.58%)	0.04978774	ko03060
HSK48/HRK48	CG	Proteasome	54 (0.79%)	0.0001072989	ko03050
		Spliceosome	189 (2.76%)	0.0003355189	ko03040
		Other types of O-glycan biosynthesis	28 (0.41%)	0.0004669705	ko00514
		Butanoate metabolism	25 (0.36%)	0.001468033	ko00650
		Porphyrin and chlorophyll metabolism	36 (0.53%)	0.002476425	ko00860
		Arginine biosynthesis	26 (0.38%)	0.008701094	ko00220
		Non-homologous end-joining	6 (0.09%)	0.009208922	ko03450
		Inositol phosphate metabolism	62 (0.90%)	0.01307215	ko00562
		One carbon pool by folate	16 (0.23%)	0.01390304	ko00670
		Fatty acid metabolism	44 (0.64%)	0.01762924	ko01212
		2-Oxocarboxylic acid metabolism	48 (0.70%)	0.03560632	ko01210
	CHG	Phosphatidylinositol signaling system	20 (1.03%)	0.04792616	ko04070
		Inositol phosphate metabolism	20 (1.03%)	0.04942088	ko00562

### Interconnection of DMGs and DEGs

To further the current understanding of the relationships between transcriptome and methylation of soybean resistance to bean pyralid larvae, the data from WGBS and RNA-Seq [10] were jointly analyzed. The correlation analysis results showed that 512 DEGs were identified as DMGs in the four comparisons, of which 265 genes showed negative regulation (Table S1), the up-regulated genes correlated with hypo-DMGs and down-regulated genes correlated with hyper-DMGs, were screened as the negatively correlated genes. In addition, 247 genes showed positive correlations, the up-regulated genes correlated with hyper-DMGs and down-regulated genes correlated with hypo-DMGs, were screened as the positively correlated genes. About 64, 93, 236 and 194 DEGs in HRK0/HRK48, HSK0/HSK48, HSK0/HRK0 and HSK48/HRK48, respectively, were associated with DMGs. There were 34, 49, 141 and 116 negatively correlated genes were identified in the four comparisons, respectively. And 11, 10, 98 and 84 negatively correlated genes in the four comparisons, respectively, were occurred in the promoter regions. Therefore, it was speculated that the changes in DNA methylation levels of 265 negatively correlated genes may be one of the reasons for the significant differences in the gene transcription levels induced by bean pyralid larvae feeding. Meanwhile, the changes in DNA methylation levels of 247 positive correlated genes may not have been the reason for the direct regulation of the gene transcription levels. Subsequently, we will focus on negatively correlated genes, which are considered to be of significance of the biological processes in plant responses to insect stimulus, whether for in-depth explorations of gene functions or pattern analyses of DNA methylation.

### KEGG enrichment analysis of negatively correlated genes

KEGG enrichment analysis of negatively correlated genes located in the gene bodies showed that (Table S2), in HRK0/HRK48, 10 negatively correlated genes (29.41%) were enriched in 11 pathways, with 4 pathways being significantly enriched, namely RNA transport, ascorbate and aldarate metabolism, fatty acid biosynthesis, and porphyrin and chlorophyll metabolism. In HSK0/HSK48, 13 negatively correlated genes (26.53%) were enriched in 23 pathways, with 5 pathways being significantly enriched, namely ubiquinone and other terpenoid-quinone biosynthesis, sulfur relay system, thiamine metabolism, selenocompound metabolism, and ether lipid metabolism. In HSK0/HRK0, 56 negatively correlated genes (39.72%) were enriched in 65 pathways, with 6 pathways being significantly enriched, namely pyruvate metabolism, propanoate metabolism, glyoxylate and dicarboxylate metabolism, carbon metabolism, lipoic acid metabolism, carotenoid biosynthesis. In HSK48/HRK48, 72 negatively correlated genes (62.07%) were enriched in 58 pathways, with 5 pathways being significantly enriched, namely carbon metabolism, propanoate metabolism, lipoic acid metabolism, pyruvate metabolism, biosynthesis of amino acids. The results suggested that various defense responses would be activated when soybean were subjected to bean pyralid larvae stress.

In addition, KEGG enrichment analysis of negatively correlated genes located in the promoter regions showed that (Table S3), in HRK0/HRK48, two negatively correlated genes (18.18%) were enriched in 4 pathways. In HSK0/HSK48, 5 negatively correlated genes (50.00%) were enriched in 10 pathways, with 2 pathways being significantly enriched, including monoterpenoid biosynthesis, and SNARE interactions in vesicular transport. In HSK0/HRK0, 30 negatively correlated genes (30.61%) were enriched in 42 pathways, with 5 pathways being significantly enriched, including endocytosis, glycolysis/gluconeogenesis, pyruvate metabolism, RNA degradation, sesquiterpenoid and triterpenoid biosynthesis. In HSK48/HRK48, 31 negatively correlated genes (36.90%) were enriched in 40 pathways, with 3 pathways being significantly enriched, including endocytosis, monoterpenoid biosynthesis, sesquiterpenoid and triterpenoid biosynthesis.

### Functional analysis of the negatively correlated genes

For further understanding the resistance mechanism of the soybean to bean pyralid larvae, Mercator software was used to obtain the classification statistics of 265 negatively correlated genes, of which 114 were annotated into 24 categories (Table S4). These genes were determined to be mainly related to such pathways as protein biosynthesis and modifications; primary metabolism; secondary metabolism; cell cycle, cell structure and component; phytohormone action; external stimuli responses, and so on.

A total of 31 DEGs related to protein metabolism and modification (Table S4), among them, two genes were up-regulated in HRK0/HRK48; five genes were up-regulated and five genes were down-regulated in HSK0/HSK48; three genes were up-regulated and one gene was down-regulated in HSK0/HRK0; one gene was up-regulated and three genes were down-regulated in HSK48/HRK48; six genes were up-regulated and three genes were down-regulated in HSK0/HRK0 and HSK48/HRK48; one gene was up-regulated in HRK0/HRK48 and HSK0/HSK48. 27 DEGs related to cell cycle, cell structure, and cell component, of which five genes were up-regulated in HRK0/HRK48; seven genes were up-regulated in HSK0/HSK48; nine genes were up-regulated and two genes were down-regulated in HSK0/HRK0; two genes were up-regulated and one gene was down-regulated in HSK48/HRK48; one gene was up-regulated in HSK0/HRK0 and HSK48/HRK48. It was found that some genes related to primary metabolism, a total of six DEGs related to lipid metabolism; two DEGs related to amino acid metabolism; three DEGs related to carbon metabolism; and five DEGs related to coenzyme metabolism were identified. Among them, one gene was up-regulated in HRK0/HRK48; three genes were up-regulated and two genes were down-regulated in HSK0/HSK48; three genes were up-regulated and one gene was down-regulated in HSK0/HRK0; two genes were up-regulated and two genes were down-regulated in HSK48/HRK48; one gene was up-regulated and one gene was down-regulated in HSK0/HRK0 and HSK48/HRK48. Two DEGs related to flavonoid biosynthesis were identified, of which one gene was down-regulated in HSK0/HRK0; one gene was up-regulated in HSK48/HRK48. Six DEGs related to transcription factor were identified, of which one gene was down-regulated in HRK0/HRK48; one gene was up-regulated in HSK0/HSK48; three genes were up-regulated in HSK0/HRK0; and one gene was up-regulated in HSK48/HRK48. The results indicated that DEGs regulated by the methylation were involved in many biological pathways after bean pyralid larvae feeding.

### Validation analysis of negatively correlated genes

**Fig. 3:**
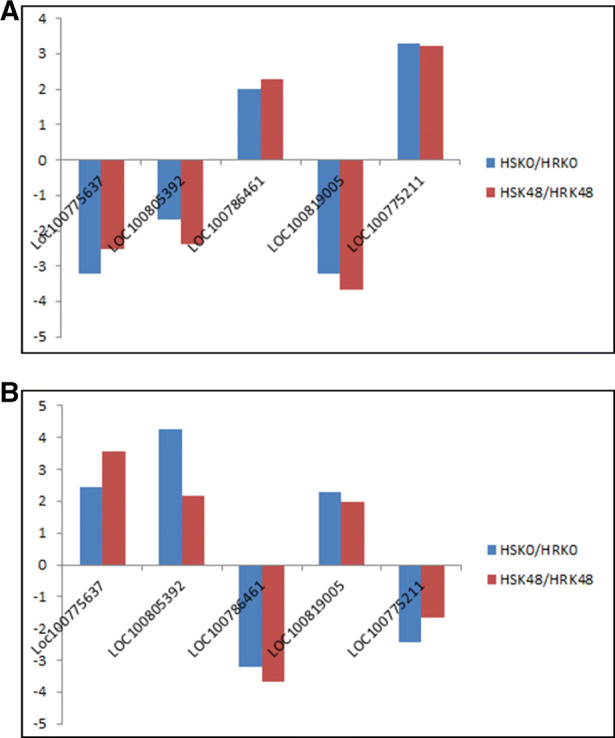
Expression levels of five DMGs validated by PS-PCR and qRT-PCR. A Differently methylated levels in HSK0/ HRK0 and HSK48/ HRK48. B qRT-PCR analysis of the genes in HSK0/ HRK0 and HSK48/ HRK48

To further verify that the negative correlations between DNA methylation and the transcriptome were not random, five negatively correlated genes were randomly selected. PS-PCR and qRT-PCR technologies were used to analyze their DNA methylation patterns and gene expression patterns. The results revealed that the PS-PCR expressed patterns and WGBS sequencing expressed patterns of five DMRs were all the same. Moreover, the qRT-PCR expression patterns of five DEGs were consistent with the RNA-Seq expression patterns (Fig. 3). These findings indicated that the sequencing results of WGBS and RNA-Seq were reliable, and that the DNA methylation may regulate the responses of soybean to pest stress by regulating the expression levels of genes related to insect resistance.

## Discussion

### Genome-wide DNA methylation characteristics of soybean resistance to bean pyralid larvae

Since DNA methylation may potentially participate in the regulations of gene expressions, as well as the maintenance of genome stability, gene silencing in plants, it thereby plays important regulatory roles in plant growth, development, and stress resistance [19, 21, 22]. We found that the genome-wide DNA methylation levels of the four samples ranged from 18.37 to 21.30%, which is consistent with the DNA methylation levels reported in other plants [23–25]. DNA methylation was found in the CG, CHG, and CHH contexts, and different contexts have different modification patterns, the methylation levels in the CG context were significantly highest than that in the CHG and CHH contexts, which suggested that the CG context was the most important methylation context in soybean. These results were consistent with the type and level of DNA methylation detected in soybean root, steam, cotyledon and leaf [26, 27]. These findings indicated that the differences of DNA methylation in all patterns may have played important roles in the soybean responses to insect stress.

### DMGs involved in regulation of soybean resistance to bean pyralid larvae

When plants are attacked by herbivorous insects, they activate a series of molecular signals in order to start their biotic defense responses [28]. By combining KEGG pathway and MapMan enrichment analysis, we found that some defense-related candidate genes had different methylation and transcription reactions after bean pyralid larvae feeding.

Protein kinases are involved in plant signaling pathways [29]. Cysteine-rich receptor-like protein kinase (CRK) and LRR-receptor-like kinase play important regulatory roles in plant growth, biotic and abiotic stresses [30, 31]. For example, 76 CRK promoter regions in soybean which contained biotic stress response elements [32]. When plants are exposed to stress, such as insect feeding, salt damage, and so on, serine/threonine protein kinase (STK) is rapidly activated by phosphorylation as serine and threonine residue. This further activates downstream signaling molecules through cascade phosphorylation for the purpose of activating specific signal transduction pathways, and finally transmits external signals to the nuclei in order to activate or inhibit the expressions of specific genes [32, 33]. The expression of STK in arabidopsis [34] and soybean [35] could be induced by insect stress. Mitogen-activated protein kinase (MAPK or MAP) is widely involved in the signal transmission of plant to stress, then activates the expression of anti-stress genes, so that plants have certain adaptability to stress [36–40]. It was also related to the defense response induced by insect stress. For example, MAPK was activated in tobacco after insect stress [41]. *NaMEK1*, *NaMEK2*, *NaMKK1*, *NaSIPKK* and *NaNPK2* in *Nicotiana attenuata* play important roles in the defense of *Manduca sexta*. They could resist plant attack by herbivorous insects [42, 43]. The growth of *Spodoptera littoralis* was inhibited by *AtMKK3* [44]. Lectin-like receptor kinases (*LecRLKs*) play important roles in plant pest resistance, they can combine with exogenous glycosyl to protect plants from insect stress [45, 46]. For example, *LecRK1* in *Nicotiana attenuata* plays an important role in plant defense responses triggered by *Manduca sexta* [47, 48]. The resistance of rice to brown planthopper (BPH) was enhanced after over-expression of *OsLecRK* [49]. *LecRK-1.8* plays an important role in the recognition of inducers derived from insect eggs in arabidopsis [50]. Our results obtained that 11 DEGs related to protein kinase, such as CRK40; CRK62; probable LRR receptor-like serine/threonine-protein kinase; STK; MAPK9; L-type lectin-domain containing receptor kinase VIII.2, and so on, were identified. It was speculated that protein kinases, as important signaling molecules, play very important regulatory roles in soybean resistance to bean pyralid larvae.

The activation and production of insect-resistant substance metabolism in plants usually consume a certain amount of growth energy, when the anti-insect defense mechanism of plants is activated, it will also lead to some changes and recombination in the morphology of the affected plants [51]. Plant cell walls are the primary cell structures sensing external stress signals, and are involved in maintaining cell morphology and related to such physiological activities as extracellular signal recognition. They are essentially the first line of defense against pathogens or pests [52–54]. Cellulose synthase complex (CSC) is assembled by cellulose synthase monomer (CesA) on the Golgi complex, and is transported by secretory vesicles and bound to cell membranes [55–57]. Plant cells can regulate cell wall formation through CSC assembly and transportation, thereby participating in plant morphogenesis and stress responses [57, 58]. It was observed that following IAA treatments of cotton, *GhCesA1* and *GhCesA2* were significantly up-regulated [59]. CSI1 is known to be involved in the formation of SmaCC/MASC and participates in the rapid recovery of CSC in plasma membrane after the stress conditions have subsided [60, 61]. Moreover, CSI1 directly mediates the interactions between CSCs and microtubules. In the absence of CSI1, the arrangements of CSCs and microtubules will be disrupted [62]. As a microfilament binding protein, fimbrin is one of the important regulatory factors of microfilament skeletons [63]. Kinesin (KIN) uses the energy produced by its hydrolysis of ATP to move along microtubules and provide power for intracellular material transport. For example, FRA1 of the arabidopsis KIN-4 family is a driver protein which moves to the positive ends of microtubules, and its function deficient mutant FRA1 showed irregular depositions of cellulose microfibrils on cell walls, making the stem brittle [64–66]. CLASP can be used as a regulatory protein of microtubule binding proteins [67, 68]. We found that a significant number of genes induced by bean pyralid larvae related to cell wall and cell cycle tissue metabolic pathways, such as CesA, CSI1, fimbrin-1, KIN-14B, KIN-14 N, KIN-4A, CLASP, and so on. The expression levels of those genes were all up-regulated after bean pyralid larvae feeding. This up-regulation may assist in the plant cell wall structuring processes in order to create a stronger physical protective layer against insects and reduce the damages to soybean undergoing insect stress, and maintain the stability of the cells and organelles. It was speculated that when soybean is subjected to pest stress, the anti-insect signaling pathways are activated after sensing cell wall damage, which activates a series of self-cell defense responses in soybean and greatly enhances the resistance of soybean. Moreover, genes related to cell cycle tissue can also effectively regulate plant tolerance to insects [69].

Cytochrome P450 (CYP) is a class of plant antioxidant inducers and detoxification genes, which can catalyze a number of substances which have defense functions in organisms, and plays an important role in the defense of organisms from diseases and insects stresses [70–72]. For example, cyanogen glycosides synthesized by *CYP79A* and *CYP71E1* in sorghum were toxic to pests [73]. The expressions of *CYP71A1* in rice [74] and *CYP51* in tobacco [75] were induced by insect stresses, thus improving plant resistance to pests. *CYP71A26* and *CYP71B34* were involved in the response to pest stress in tea plants [76]. We observed that the expression of cytochrome P450 81E8 in the resistant material was higher than that in the susceptible material after bean pyralid larvae feeding. The results indicated that the release of terpenoids from the resistant material could be induced by pest stress. It was speculated that soybean can utilize cytochrome P450 family to reduce the threats caused by pests.

Transcription factors can regulate the expressions of multiple genes related to biotic stress, and improve the resistance of plant to disease and insects [77, 78]. ERF transcription factor plays an important regulatory role in plant resistance to insect infestations [79]. For example, the combination of *SSaERF1* and GCC-box can enhance the resistance of arabidopsis to *Prodenia litura* [80], and *BrERF11b* can enhance the resistance of tobacco to *Myzus persicae* and *Prodenia litur*a [81], thus indicating that plant resistance to insect stress can be improved through the over-expression of ERF. BEE1, bHLH and GATA transcription factors play important role in plant resistance to biotic and abiotic stresses [82, 83]. Before bean pyralid larvae feeding, the expression levels of ERF and BEE1 in the resistant material were higher than those in the susceptible material, which indicated that these two transcription factors were related to the genotypes of the resistant material. After insect stress, bHLH25 and GATA 26 were induced in the resistant material, and bHLH79 was induced in the susceptible material. Therefore, it was speculated that the differential expressions of these transcription factors may be an important reason for the differences of induced resistance levels and the persistence of resistant and susceptible soybean varieties.

## Conclusions

In order to further understand the molecular mechanism of soybean responses to bean pyralid larvae, we used WGBS to analyze the genome-wide methylation of highly resistant and highly susceptible soybean leaves before and after bean pyralid larvae feeding. It was found that DNA methylation levels of specific genes changed in response to insect stress. At the same time, according to the DNA methylation and transcriptome association analysis, we concluded that there was a mainly negative correlation between DNA methylation and gene expression to a certain extent. In addition, the response to bean pyralid may be related to the pathways, such as protein biosynthesis and modification; primary and secondary metabolisms; cell cycle, cell structure and component; phytohormone action; RNA biosynthesis and processing, and so on. Meanwhile, by analyzing the expression levels and DNA methylation levels of those genes, the relationships between their methylation status and expression levels in different materials were revealed, and the roles of these related genes in the induction processes could be explored. This research investigation comprehensively analyzed the molecular mechanism of soybean undergoing insect stress from the transcription levels and methylation levels, which was of great significance to the research of soybean insect resistance.

## Materials and method

### Experimental materials

Both the highly resistant material (Gantai-2-2) and highly susceptible material (Wan 82–178)(Fig. S1) were planted in gray insect-proof net room on a test field at the Guangxi Academy of Agricultural Sciences in Nanning, Guangxi, China. When the plant growth reached 10 compound leaves, the fourth instar larvae of bean pyralid were grafted to each seedling according to a density of five larvae. Samples were taken at 0 h and 48 h after grafting. The samples were quickly frozen in liquid nitrogen and stored at − 80 °C for further use.

### Total DNA extraction and detection

Total genomic DNA was extracted from soybean leaves using a DNeasy Plant Mini Kit (Qiagen, Valencia, CA, USA). The degradation of DNA in the samples was detected by agarose gel electrophoresis. The OD260/280 values of DNA were detected using a Nano Drop 2000 spectrophotometer (Thermo Fisher Scientific, MA, USA), and the concentration levels of DNA were accurately quantified by Qubit Fluorometer (Invitrogen, CA, USA). The qualified DNA samples were used for the library construction.

### Sequencing analysis of the bisulfite

For WGBS librarys constructing, the DNAs were broken into fragments with a mean size of 250 bp using Bioruptor (Diagenode, Belgium). Following end repair and adenylation, the sonicated DNA fragments were ligated to cytosine-methylated barcodes according to manufacturer’s instruction. The DNA fragments were treated with bisulfite using the ZYMO EZ DNA Methylation-Gold kit (Zymo research, Orange County, CA, USA). Different *Insert size* fragments were excised from the same lane of a 2% TAE agarose gel. The products were purified by using a QIAquick Gel Extraction kit (Qiagen, Valencia, CA, USA) and then amplified by PCR. Finally, the qualified DNA libraries were sequenced on the Illumina Hiseq4000 platform (BGI-Shenzhen, Shenzhen, China).

### Data filtering and sequence alignment

The raw data were filtered by removing adapter sequences, contamination and low-quality reads. After the filtering process was completed, BSMAP software [84] was used to map the clean reads with the soybean reference genome (https://www.ncbi.nlm.nih.gov/assembly/GCF_000004515.4), and the comparison rates and bisulfite conversion rates were calculated. In order to calculate the methylation levels of each site, we calculated the proportion of the number of reads supporting methylation to the total number of reads covering the site [85]. The formula was as follows: 

Where Nm and Nn represent the reads number of mC and nonmethylation-C, respectively.

### DMR detection

A window containing at least five CG (CHG or CHH) was found at the same position in two of the sample genomes, and the differences in the CG methylation levels between the two samples of that window were compared. The region with significant differences (Fisher’s Exact, 2-fold change, and *p*-value ≤0.05) in the methylation between the two samples was referred to as DMR. If the contiguous region formed by the two adjacent DMRs differed significantly in methylation levels in the two samples, the two DMRs were combined into a single contiguous DMR. Otherwise, they were considered to be two independent DMRs.

CIRCOS software was used to compare the methylation levels of DMR in the different samples [86]. The degree of difference of a methyl-cytosine (mCG, mCHG, mCHH) was also calculated using the following formula: .

Where *Rm1* and *Rm2* represent the methylation levels of mC for sample 1 and sample 2, respectively. If the value of *Rm1* or *Rm2* is 0, it shall be replaced by 0.001.

### GO and KEGG analysis

Gene Ontology (GO) enrichment analysis method was used to provide all the GO terms which were significantly enriched in the DMGs, and to filter the DMGs with specific biological functions. Based on the GO TermFinder (http://www.yeastgenome.org/help/analyze/go-term-finder) [87], the number of genes in each term was calculated. Then, a hypergeometric test method was used to find the GO terms which were significantly enriched in the DMGs when compared with the entire genome background. The GO terms with a *p*-value≤0.05 were regarded as significantly enriched.

KEGG is the main public database for those pathways [88]. Through significant enrichment analyses of the pathways, it can be determined which pathways are significantly enriched in the DMGs when compared with the whole genome background, taking the KEGG pathway as a unit. Pathways with a *p*-value≤0.05 were regarded as significantly enriched.

### Conjoint analysis of genome-wide DNA methylation and transcriptome

The original data obtained from WGBS and RNA-Seq [10] were analyzed and compared. The intersections of DNA methylation levels and gene expression levels were taken for conjoint analysis, and the DEGs in DMGs were screened out. The correlation between the methylation level of DMR and the expression level of DEG was detected by pearson correlation analysis. There were five overlapping situations involved: DMR_genes_VS_DEG_genes; DMR_Hypergenes_VS_DEG_upgenes; DMR_Hypergenes_VS_DEG_downgenes; DMR_Hypogenes_VS_DEG_upgenes; DMR_Hypogenes_VS_DEG_downgenes. The criterion for selecting the intersection genes were *p*-value < 0.05 [20].

### MapMan biological function annotation

The amino acid sequence of the unigene coding protein obtained by CDS analysis was submitted to the MapMan website application online software mercator (http://mapman.gabipd.org/web/guest/mercator) for annotation of the biological functions of the encoding protein. The mapping information of the biological processes of the species was obtained.

### Pyrosequencing PCR (PS-PCR) validation

Five genes with negative correlations between DNA methylation and gene expression were randomly selected. DNA methylation validation was conducted using the PS-PCR method. The DMR regions corresponding to those five genes were identified. All primers were designed using PyroMark Assay Design 2.0 (Table S5) and commercially synthesized (BGI, Shenzhen, China). The PCR was conducted in the following conditions: a total volume of 50 μL, containing 10.0 μL 5× buffer GC (KAPA), 1.0 μL dNTP, 1.0 μL of each primer, 2.0 μL template, 0.2 μL Taq Master Mix and 34.8 μL ddH_2_O. The thermal cycling conditions were as follows: 95 °C for 3 m; followed by 40 cycles of heating at 94 °C for 30 s, 50 °C for 30 s, 72 °C for 1 m and annealing at 72 °C for 7 m.

### Quantitative real-time PCR (qRT-PCR) validation

The primer sequence was designed with Primer Premier 5.0 software (Premier Biosoft International, Palo Alto, CA) (Table S5). Next, 1.0 μg of total RNA was reverse-transcribed by reverse transcriptase according to the protocol of iScript cDNA Synthesis Kit (Bio-Rad, CA, USA), and used as the template for the following qRT-PCR amplification. The qRT-PCR reaction mixture (25.0 μL) contained 10.0 μL SybrGreen qPCR Master Mix (2× concentration, Ruian Biotechnologies, Shanghai, China), 0.6 μL upstream primer, 0.6 μL downstream primer (10.0 μM), 1.0 μL cDNA, and 7.8 μL ddH_2_O. The thermal cycling conditions were as follows: pre-denaturation at 95 °C for 2 m; followed by 40 cycles of heating at 95 °C for 10 s and annealing at 60 °C for 40 s. *β*-actin gene was used as the internal control gene. The relative level of genes expression was evaluated by the 2^*-ΔΔct*^ method.

## Supplementary Information

**Additional file 1: Table S1** The negatively correlated genes between transcription and methylation of soybean resistance to bean pyralid larvae.

**Additional file 2: Table S2** Pathway analysis of the negatively correlated genes in the gene bodies.

**Additional file 3: Table S3** Pathway analysis of the negatively correlated genes in the promoter regions.

**Additional file 4: Table S4** MapMan cluster analysis of the negatively correlated genes.

**Additional file 5: Table S5** Primers for the qRT-PCR and PS-PCR.

**Additional file 6: Fig. S1** The resistant material and susceptible material under bean pyralid larvae feeding for 48 h. A: Gantai-2-2; B: Wan82–178.

## Data Availability

All data were submitted to the National Center for Biotechnology Information (NCBI) under SRA number SRA549176.

## Abbreviations

WGBS: Whole-genome bisulfite sequencing
RNA-Seq: RNA-sequencing
DMG: Differentially methylated gene
DMR: Differentially methylated region
DMP: Differentially methylated promoter
DEG: Differentially expressed gene
GO: Gene ontology
KEGG: Kyoto encyclopedia of genes and genomes
PS-PCR: Pyrosequencing PCR
qRT-PCR: Quantitative real-time PCR
CRK: Cysteine-rich receptor-like protein kinase
STK: Serine/threonine protein kinase
CSC: Cellulose synthase complex
CesA: Cellulose synthase monomer
KIN: Kinesin
SBT: Subtilisin-like protease
CYP: Cytochrome P450
