# Correction to: Methylome and transcriptome analyses of soybean response to bean pyralid larvae

**DOI:** 10.1186/s12864-021-08252-3

**Published:** 2021-12-22

**Authors:** Wei-Ying Zeng, Yu-Rong Tan, Sheng-Feng Long, Zu-Dong Sun, Zhen-Guang Lai, Shou-Zhen Yang, Huai-Zhu Chen, Xia-Yan Qing

**Affiliations:** Guangxi Academy of Agricultural Sciences, Nanning, 530007 Guangxi China

**Correction to: BMC Genomics 22, 836 (2021)**

**https://doi.org/10.1186/s12864-021-08140-w**

Following publication of the original article [1] it was reported that the authorship panel listed too many corresponding authors. Zu-Dong Sun is the sole corresponding author. The authorship list has been updated and the original article has been corrected.
