# Supplementary material for: Methylome and transcriptome analyses of soybean response to bean pyralid larvae
Source: BMC Genomics. 2021 Nov 18;22:836. doi: 10.1186/s12864-021-08140-w (PMC8603512; doi:10.1186/s12864-021-08140-w)
Supplement: Supplementary file 5 — Additional file 5: Table S5 Primers for the qRT-PCR and PS-PCR. [file 12864_2021_8140_MOESM5_ESM.docx]

Table S5 Primers for the qRT-PCR and PS-PCR

|  | Gene | Forward | | | Reverse |
| --- | --- | --- | --- | --- | --- |
| qRT-PCR | LOC100775637 | | CCTACCTCCACTGCCAGCATCTC | CCAATAGGGCGTGCCGACAACA | |
|  | LOC100805392 | | CTTAGTGAATTAACTTACAACCCAACC | ATAGCCTACCAAGACATAAACGAGA | |
|  | LOC100786461 | | CACCATTTCCCACTTCCCATTAC | GACCCTTCATTGAGAACCTTTGC | |
|  | LOC100819005 | | GTTTCCGCCGTATCCATTCAGTC | ATAAACCACTCCAAATCCACCTT | |
|  | LOC100775211 | | TTCTGGTGGCAGGGAAGGTTCTG | GCAGCCTGGGTACTGGCATTGTT | |
| PS-PCR | LOC100775637 | | AGGTGAGAGAGAGGAGGTTTTTTAGAGGGATGGGGTTTTGGTT | ACATCCTCATTTACACCAATAAAC CCCCTTCCTATATACCCCTTTATAAATTCC | |
|  |  |  |  |  | |
|  | LOC100805392 | | AGTGGTTTGGAATTAGGTTAAGG | ATTCTCATTCTTAATATCAACCCATAAATT | |
|  | LOC100786461 | | GTTTTGGTTGTATGTTGTGGTTTAA | CAACTTCCTCACCCTAATTTAATCTCTC | |
|  | LOC100819005 | | TGGTTATATGATGATGTAAATGGTAGTAG | AATCATCACTTCCACCATCAATTAT | |
|  | LOC100775211 | | GTTAAAAAGGAAATTTATAGTGATTTTG | ACTCTCTTTCCATTCAAAAATTACTATT | |
