# Supplementary figures and images for: Methylome and transcriptome analyses of soybean response to bean pyralid larvae
Source: BMC Genomics. 2021 Nov 18;22:836. doi: 10.1186/s12864-021-08140-w (PMC8603512; doi:10.1186/s12864-021-08140-w)

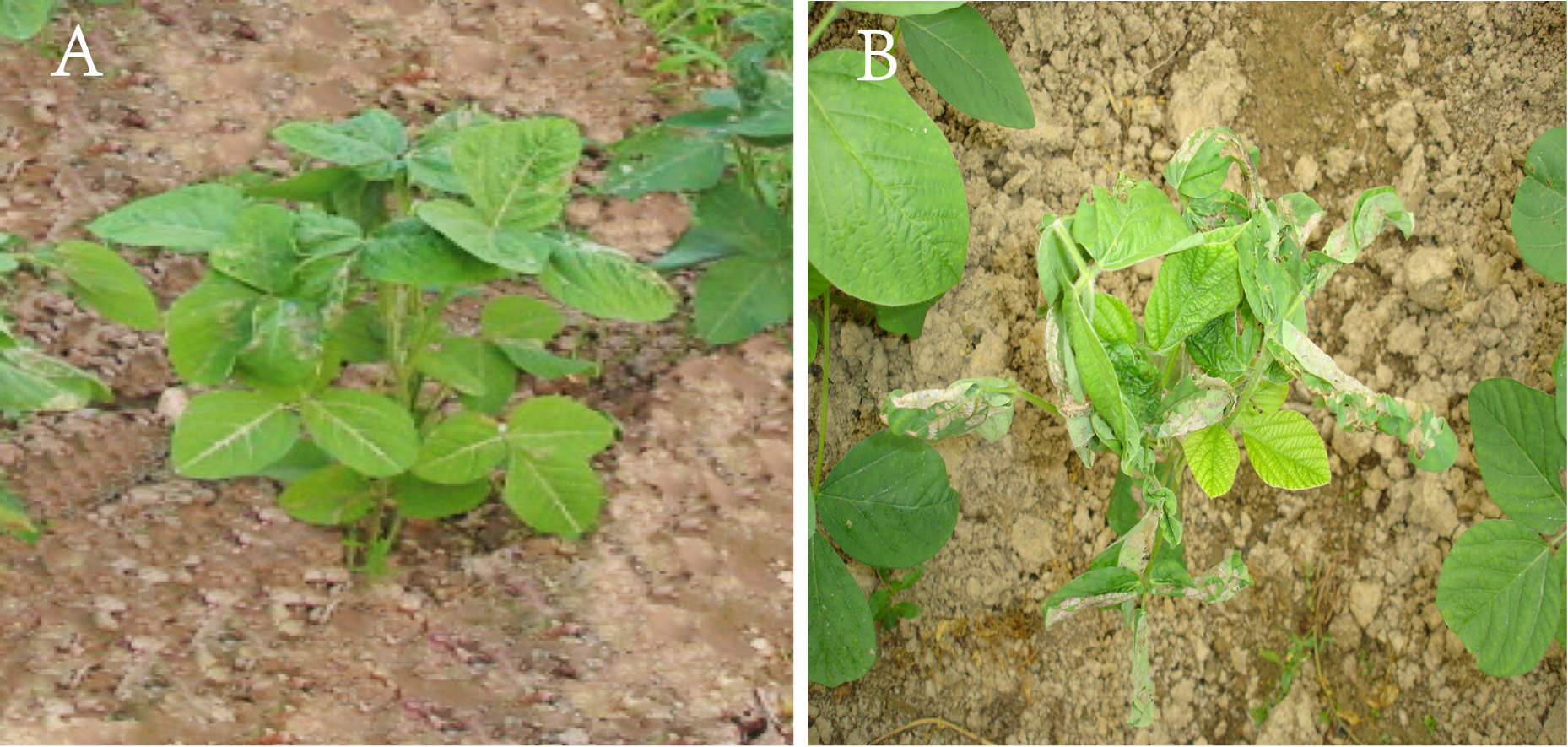

Supplement: Supplementary file 6 — Additional file 6: Fig. S1 The resistant material and susceptible material under bean pyralid larvae feeding for 48 h. A: Gantai-2-2; B: Wan82–178. [file 12864_2021_8140_MOESM6_ESM.tif]
